# Effectiveness of malaria chemoprevention in the first two years of life in Cameroon and Côte d’Ivoire compared to standard of care: study protocol for a population-based prospective cohort impact evaluation study

**DOI:** 10.1186/s12889-024-19887-8

**Published:** 2024-09-06

**Authors:** Gillian Stresman, Sham Lal, Jane Bruce, Akindeh Nji, Assi Serge-Brice, Jonna Mosoff, Alba McGirr, Georgia Gore-Langton, Michaela McGuire, James Sinsai, Albertine Lele, Mercy Tah-Monunde, Zah-Bi Kouadio, Mian Anatole, Abibatou Konate-Toure, Sian Elisabeth Clarke, Roland Gosling, Wilfred Fon Mbacham, William Yavo, R. Matthew Chico

**Affiliations:** 1 Department of Epidemiology, College of Public Health, University of South Florida, 13201 Bruce B Downs Blvd, Tampa, FL USA; 2 Department of Infection Biology, Faculty of Infectious and Tropical Diseases, London School of Hygiene & Tropical Medicine, Keppel Street, London, UK; 3 Department of Disease Control, Faculty of Infectious and Tropical Diseases, London School of Hygiene & Tropical Medicine, Keppel Street, London, UK; 4 Fobang Institute for Innovation in Science and Technology, 14 Missionary Road, Simbok, Yaounde, Cameroon; 5 Department of Biochemistry, Faculty of Sciences, The University of Yaounde I, Yaounde, Cameroon; 6 National Institute of Public Health, B.P. V47, Abidjan, Côte d’Ivoire; 7 UFR of Pharmaceutical and Biological Sciences, Department of Parasitology-Mycology, Félix Houphouët-Boigny University, Abidjan, B.P. V34 Côte d’Ivoire; 8 Department of Epidemiology and Biostatistics,, University of California San Francisco, San Francisco California, USA

**Keywords:** Perennial malaria chemoprevention, Intermittent preventive treatment, Burden reduction, Cohort stud, Early childhood

## Abstract

**Background:**

Perennial malaria chemoprevention (PMC) is a chemoprevention strategy endorsed by the World Health Organization (WHO) and is increasingly being adopted by National Malaria Programmes. PMC aims to reduce morbidity and mortality caused by malaria and anaemia in in young children through provision of antimalarial drugs at routine contact points with the local health system. This study aims to evaluate the impact of the programmatically-implemented country-tailored PMC programmes targeting children up to two years of age using sulfadoxine-pyrimethamine (SP) on the incidence of malaria and anaemia in children in Cameroon and Côte d’Ivoire.

**Methods:**

We will assess the impact of PMC using passive and active monitoring of a prospective observational cohort of children up to 36 months of age at recruitment in selected study sites in Cameroon and Côte d’Ivoire. The primary and secondary outcomes include malaria, anaemia and malnutrition incidence. We will also conduct a time-series analysis of passively detected malaria and anaemia cases comparing the periods before and after PMC introduction. This study is powered to detect a 30% and 40% reduction of malaria incidence compared to the standard of care in Cameroon and Côte d’Ivoire, respectively.

**Discussion:**

This multi-country study aims to provide evidence of the effectiveness of PMC targeting children in the first two years of life on malaria and anaemia and will provide important information to inform optimal operationalization and evaluation of this strategy.

**Trial Registration:**

Cameroon - NCT05889052; Côte d’Ivoire - NCT05856357.

**Supplementary Information:**

The online version contains supplementary material available at 10.1186/s12889-024-19887-8.

## Background

Despite impressive reductions over the past 15 years, the burden of malaria-attributable morbidity and mortality has increased recently from an estimated 219 million malaria cases in 2019 to 249 million cases in 2022 [1–3]. Malaria deaths have followed the same trend with an increase from an estimated 568,000 in 2019 to 619,000 in 2021. Nearly all (96%) of these deaths occurred in 29 African countries. Although children under five years of age are most at risk of dying from malaria, children under two are estimated to account for one-third (36%) of global malaria deaths [2, 4].

In 2010, the World Health Organization (WHO) recommended the provision of three-doses of sulfadoxine-pyrimethamine (SP) in the first year of life, an intervention known as intermittent preventative treatment for malaria in infants (IPTi). A Cochrane review of placebo-controlled randomized controlled trials of IPTi showed the intervention reduced clinical malaria by 30% (rate ratio [RR] 0.70, 95% confidence interval (CI) 0.62 to 0.88), anaemia by 18% (RR 0.82, 95% CI 0.68 to 0.98), and parasitaemia by 34% (RR of 0.66, 95% CI: 0.56–0.79) [5]. However, parasite resistance to SP has undermined confidence in the use of this drug for national policy: This might explain why only one malaria-endemic country, Sierra Leone, has adopted IPTi over the past decade [6]. Since the initial WHO recommendation, new data suggest that expanding the number of doses of SP, as well as increasing the age of eligible children beyond the first year of life may confer enhanced protection [2, 7, 8]. Prompted by these findings in 2022, the WHO recommended the provision of perennial malaria chemoprevention (PMC) [9, 10]. The PMC intervention involves the administration of a full treatment-course of an antimalarial drug at predefined age intervals, regardless of infection, as a chemoprevention strategy in areas with high perennial transmission. Per WHO guidelines, the PMC schedule should be informed by the age pattern of severe malaria cases, the duration of protection of the selected antimalarial, and the feasibility and affordability of delivering each additional PMC course [9]. However, to understand whether investing in PMC represents an effective use of scarce resources, rigorous evidence on its potential effectiveness at reducing malaria and anaemia incidence is needed.

### Overview of PMC intervention

As part of a Unitaid funded project – the Plus Project – Cameroon and Côte d’Ivoire National Malaria Programmes (NMPs) opted to join a process of designing, implementing, and evaluating a pilot PMC project. Other evaluations taking place concurrently, but not reported here, include a cost effectiveness analysis, drug resistance monitoring, a process evaluation, and policy adoption. Briefly, in both countries, the interventions were developed through a co-design process involving key stakeholders, including malaria and vaccination programmes, local leaders and other community representatives, that decided on the number of PMC doses and when to administer them as part of routine health services, as well as and the geographic areas for implementation [11]. The intervention was restricted to the use of SP, with a maximum of eight doses over the first two years of life. Delivery channels and timings of doses were under the control of country stakeholders.

**Fig. 1:**
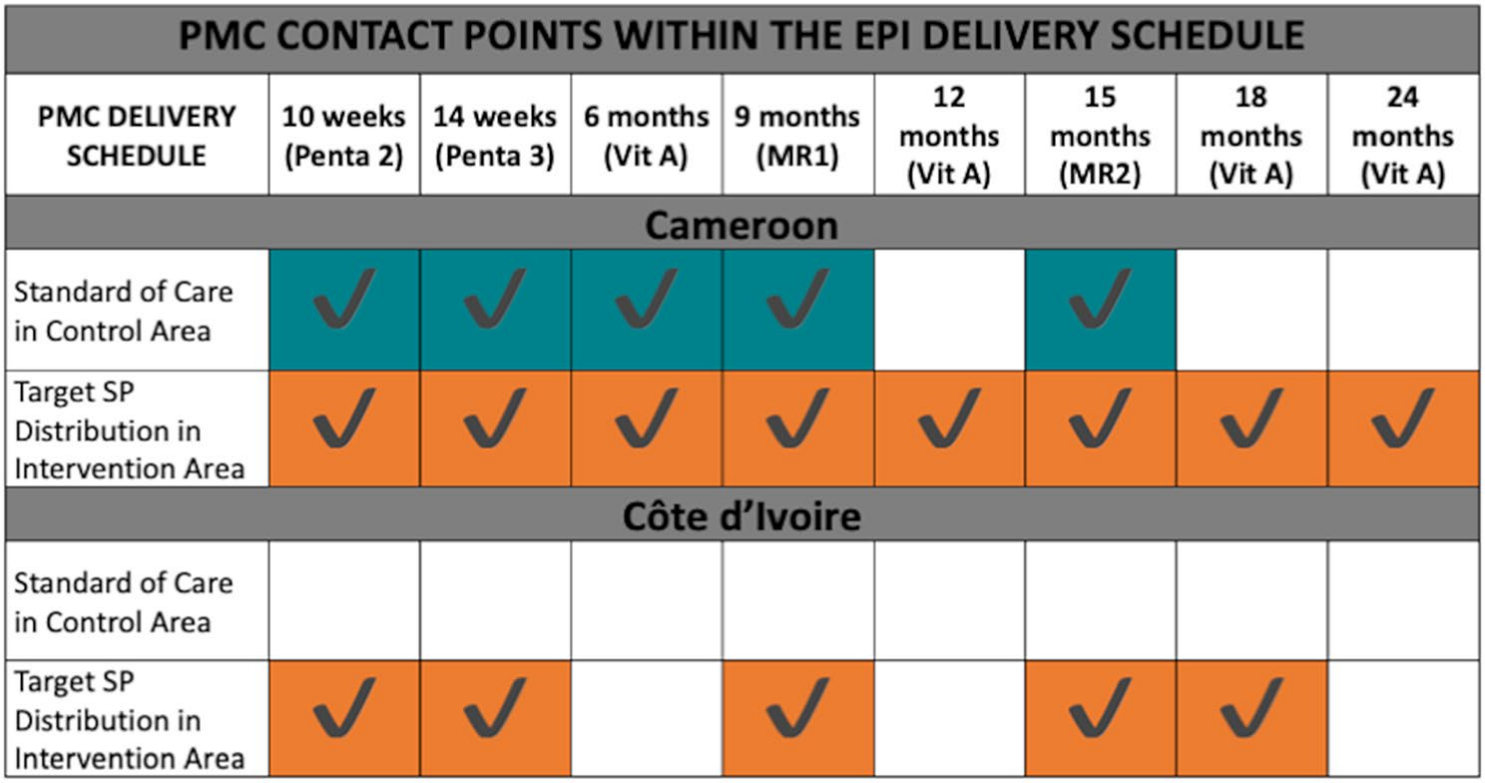
Title: Perennial malaria chemoprevention (PMC) dose schedule in intervention and areas receiving standard of care as integrated within the existing essential programme on immunization (EPI) delivery schedule Legend: Perennial malaria chemoprevention (PMC) delivery schedules in Cameroon and Côte d’Ivoire denoted by black checkmarks with the Plus Project activities denoted in orange and any doses of PMC as part of standard of care shown in orange alongside vaccination visits and ideal child age as part of the Essential Programme on Immunization (EPI) and the standard of care outlined in national guidelines. Note: Penta 2 and penta 3 refer to the 2nd and 3rd dose of the pentavalent vaccine providing protection against diphtheria, pertussis, tetanus, hepatitis B and haemophilus influenza B. Vit A refers to when doses of vitamin A are scheduled. MR1 and MR2 refers to vaccination against measles and rubella

Cameroon decided on an eight-dose PMC schedule whereas Côte d’Ivoire opted for a five-dose approach, both leveraging visits in the current Essential Programme of Immunization (EPI) and Vitamin A delivery schedule. The standard of care in Cameroon and Côte d’Ivoire is five and zero SP doses, respectively (Fig. 1). SP will be delivered at health facilities using EPI infrastructure, by community health workers (CHWs), and actively targeting populations where vaccination coverage is low. All PMC will be delivered by the Ministry of Health (MoH) in each country and will prioritize areas (i) with the highest malaria incidence and (ii) where other chemoprevention-based interventions are not implemented. Eligibility for children to receive PMC will be assessed by the MoH staff at the point of SP administration. PMC eligibility includes being the correct age to receive each dose scheduled in concordance with the EPI schedule, not having received SP within the previous four weeks, no known allergies to SP, or not concurrently taking any contraindicated medication according to manufacturer’s guidelines. All children will be eligible to receive PMC irrespective of whether they participate in the study activities.

### Aims and objectives

This is a multi-site study with the primary aim to evaluate the impact of routine delivery of PMC on malaria incidence during the first two years of life. In Cameroon we will compare the effect of up to eight versus the standard of care of five doses, whilst in Côte d’Ivoire we will compare the effect of five versus zero SP doses, as the standard of care. Secondary objectives include determining any differences in: (i) severe malaria, malarial deaths, anaemia, and severe anaemia; (ii) differences in all indicators by country, delivery platform and the number and timing of doses; and (iii) any dose response effect or differences according to distance to the health facilities, as a proxy for access to care.

Evaluation of malaria interventions requires knowledge of the regional epidemiology of malaria and active and passive surveillance methods have been proposed for malaria vaccine efficacy studies. Passive case detection methods are limited to those seeking health care and are likely to underestimate burden of malaria in the community but may provide information on the clinical presentation and management of malaria relevant for planning health services. In contrast, active surveillance methods can minimise the effect of health care-seeking behaviour on malaria detection and measures of effect. It also allows for understanding the age, spatial and temporal patterns of disease in community settings. To address these issues, we will employ two approaches to address the study objectives. First, we will develop a passive cohort, which will capture routinely collected health data as part of all child visits to participating health facilities in the selected study sites. Secondly, we will form and prospectively follow an active cohort of children who will be visited at their households every three months over the study period. Reported malaria and anaemia cases in children under 5 years from the 5 years prior to the start of the study will also be collected to ensure results can be interpreted within the context of the broader malaria transmission patterns at each site.

## Methods

### Study site

**Fig. 2:**
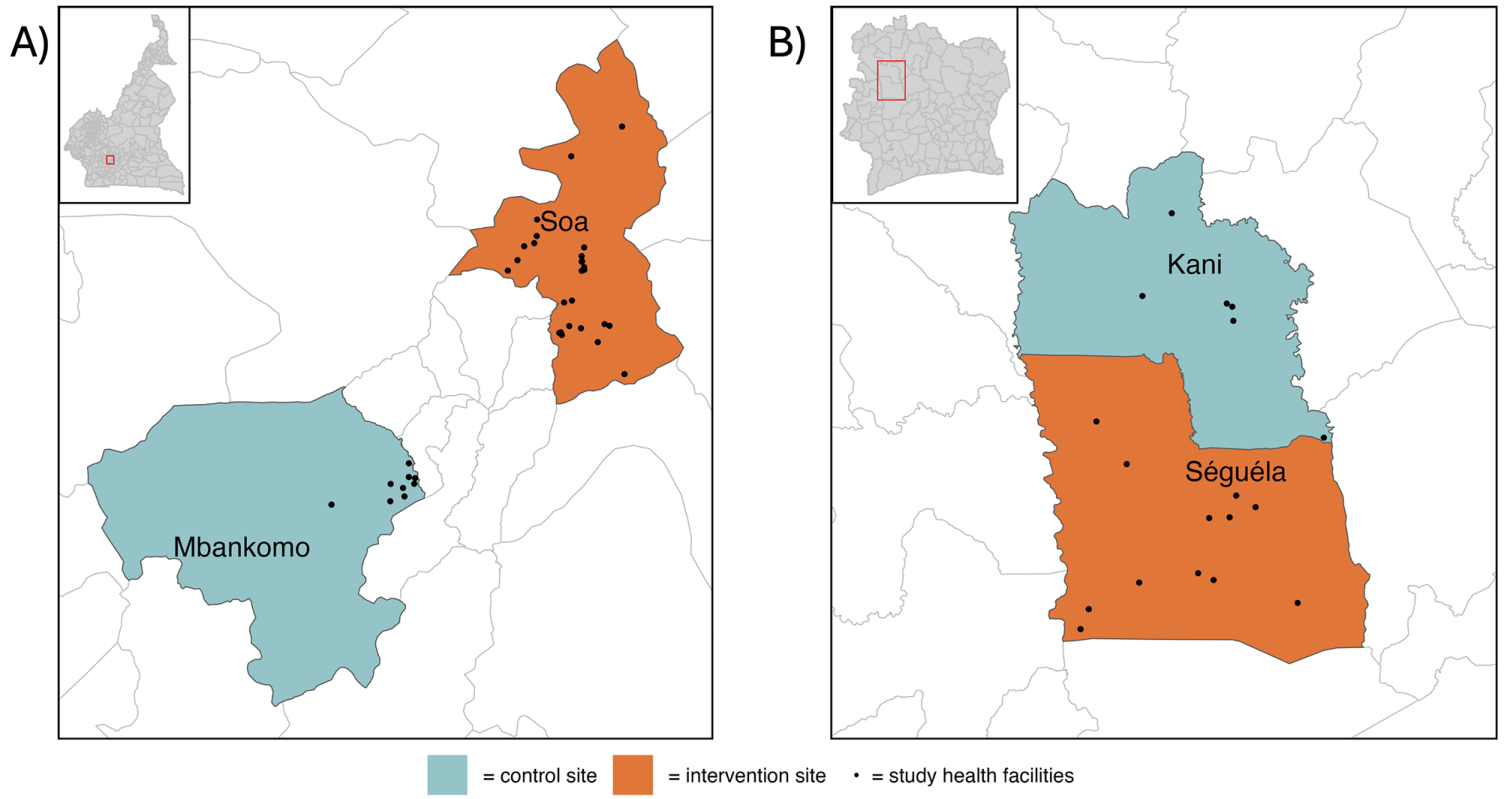
Title: Map of study areas. Legend: Study districts including, the intervention (orange) and control areas (teal) in the Central Region in Cameroon (**A**) and in the Worodougou Region in Côte d’Ivoire (**B**), are shown. Selected health facilities are represented by the black dots. Study regions within each country are shown in the red bounded areas in the inset maps

Areas with the highest malaria transmission in both Cameroon and Côte d’Ivoire were purposively selected to be PMC implementation areas to maximize any observable impact of the intervention. Control areas in both countries were also identified that had epidemiological profiles that were similar to the intervention areas. In Cameroon, we chose Soa and Mbankomo in the Central Region as the intervention and the control areas, respectively, whilst in Côte d’Ivoire, Séguéla and Kani in the Worodougou Region were chosen as the intervention and control areas, respectively. These sites were chosen based on the size of the population, number of EPI facilities, malaria incidence, EPI coverage, and availability of a suitable control population receiving standard of care (Fig. 2).

### Cameroon

Soa and Mbankomo are peri-urban areas located on the north-eastern and south-western periphery of Yaoundé, respectively (Fig. 2). Both areas have an estimated population density of 1,235 people per squared km and 15% of the population is under 5 years of age. In total, there are 39 health facilities in Soa, 12 of which are public and 28 have active CHWs. Of the 39 facilities, 20 administer EPI services and are eligible for SP administration as part of the expanded PMC programme. In Mbankomo, there are 32 health facilities, of which 4 are public and 10 have active CHWs. Of the 32 facilities, 19 administer EPI services and are eligible for SP administration as part of the standard of care PMC programme. Malaria incidence in both Soa and Mbankomo was 597 cases per 1000 children under 5 in 2020 [12]. Malaria transmission is typically highest during the two rainy seasons, from March to June and from October to November.

### Côte d’Ivoire

Séguéla and Kani are rural areas located approximately 500 km northwest of the capital city, Abidjan (Fig. 2). The estimated population density is 45.98 people per squared km (2021) and 18% of the population is under 5 years of age. In total, there are 42 health facilities in Séguéla, all of which are public and 33 of these have active CHWs. Of the 42 facilities, 41 administer EPI services and thus are eligible for SP administration as part of the expanded PMC programme. In Kani, there are 8 health facilities, all of which are public and have active CHWs. Of the 8 facilities, all administer EPI services and thus would be eligible for SP administration if any doses were given as part of a standard of care PMC programme. According to the NMP, malaria incidence in both Séguéla and Kani was 572 cases per 1000 children under 5 in 2020 with the peak typically from April to July during the rainy season.

### Study design

**Fig. 3:**
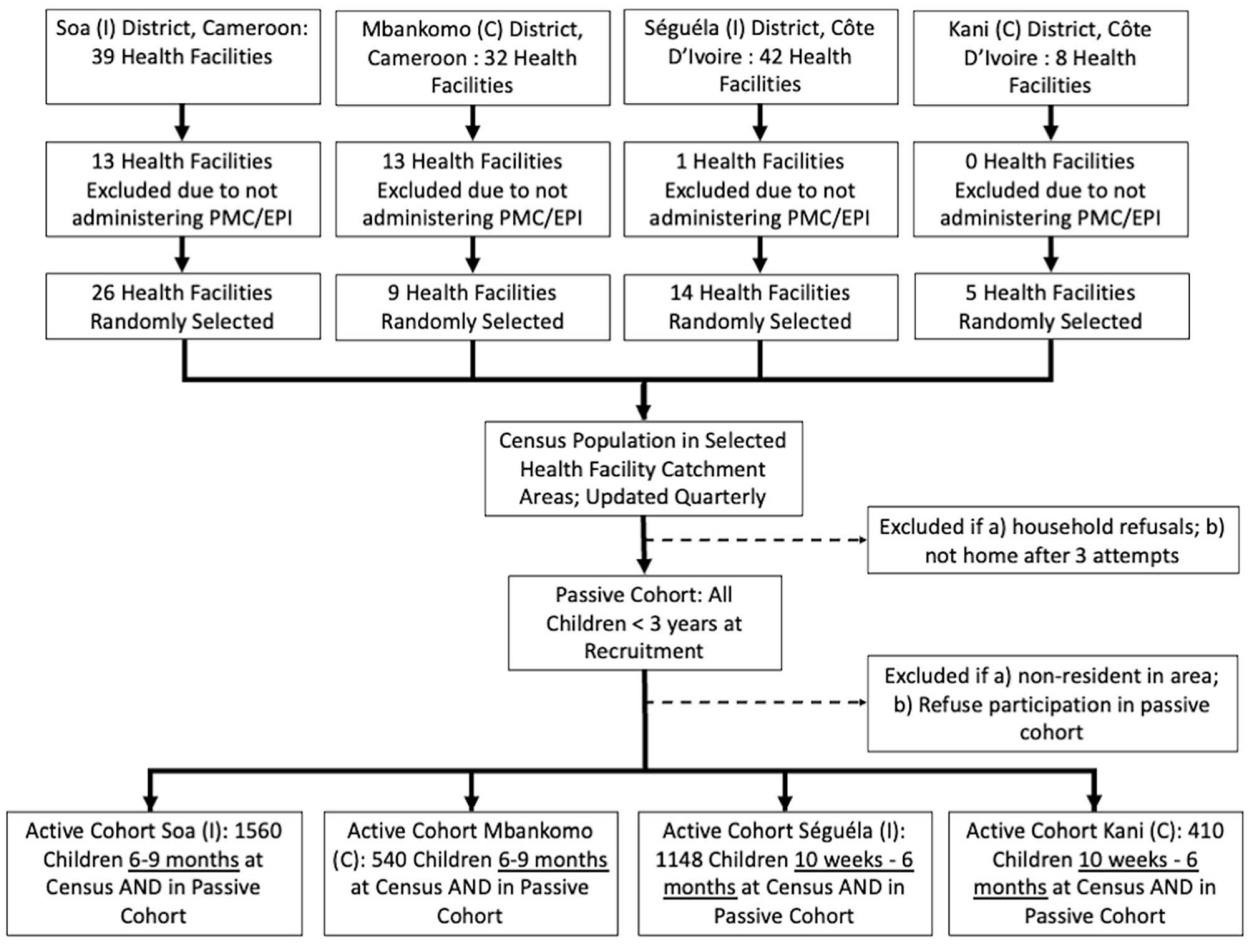
Title: Study population Legend: Overview of the structure of the study design including sample size of facilities and children for each component and how many were excluded at the design stage

All health facilities providing EPI services in selected study sites and corresponding catchment populations will be eligible for inclusion and constitute the primary sampling unit for this evaluation. Health facilities will be randomly selected for inclusion (Fig. 3). All non-EPI/PMC facilities within the selected EPI catchment areas, will be included in the passive cohort data collection, described below (Table 1). In the selected EPI facility catchment areas, several study activities will be conducted: (i) a census to enumerate all households, primary caregivers and children under 36 to characterize the population eligible to receive PMC (0–24 months) as well as when they age out of the intervention (25–36 months), (ii) a passive cohort at health facilities to monitor clinical episodes of malaria and anaemia as well as receipt of EPI vaccinations and PMC in children under 36 months, and (iii) to monitor malaria and anaemia in a subgroup of participating children in an active cohort over an 18-month period. The same procedures will be employed in both intervention and control areas in Cameroon and Côte d’Ivoire.

**Table 1:** Eligibility criteria for each study component in the PMC impact evaluation study

	Inclusion Criteria	Exclusion Criteria
**Health Facilities**	- Located within the target study sites - Administer EPI/PMC services	- Located outside of the target study area
**Census - Household**	- Located within the catchment area of selected health facilities - Head of household provides consent	- Located outside of the selected health facility catchment area - Head of household not present or refuses participation
**Passive Cohort - Child**	- Regularly resides in households included in the census - Age 36 months or less at time of census - Primary caregiver provides informed consent	- Head of household refused participation in the census - Primary caregiver not present or refuses participation - Not expected to reside in the selected household for the study duration - Age greater than 36 months of age at recruitment visit
**Active Cohort - Child**	- Recruited into the passive cohort - Aged between 6–9 months in Cameroon and 10 weeks and 6 months in Côte d’Ivoire at point of recruitment - Primary caregiver provides informed consent	- Head of household/caregiver refused participation in the census and/or passive cohort - Not within the identified age category at the point of recruitment - Primary caregiver not present or refuses participation

### Census

A census of the population will be conducted in each selected EPI catchment area to create a sampling frame that will support both the passive and active cohorts. Specifically, all households in the selected EPI catchment areas will be approached for inclusion (Table 1). Written, informed consent will be obtained from the head of household for participation in the study. Information recorded will include the GPS coordinates of the household, information on house construction, socioeconomic position, use of vector control interventions, healthcare-seeking behaviours, and the number of people residing in the household composition according to age groups. (i) ≤ 3 years, (ii) > 3 to < 5 years, (iii) ≥ 5 years to < 21 years, and (iv) ≥ to 21 years. Each household will be assigned a unique, non-identifiable number that will be included on study documentation and written on household’s doorframe for future localisation.

The census will take place prior to the PMC implementation and will be updated at 3-monthly intervals throughout the 18-month study period. During the census update, any births, deaths, or immigration and emigration will be recorded. The same study procedures during the initial census visit will be followed for new recruits. For any participants that have died since the previous visit, a brief questionnaire will be administered where possible to obtain information on the date of death, whether it was in a health facility or at home, and broad categories of the potential cause of death (i.e., traffic accident, health episode etc.) and other relevant information. Due to the sensitive nature of this data and difficulty in obtaining the precise cause of death in the community, verbal autopsies will not be conducted and any deaths without a confirmed cause of death will be excluded from analysis.

### Passive cohort

All children up to 36 months of age residing in enumerated households during the census will be eligible for inclusion in the passive cohort and enrolled upon provision of written informed consent from their primary caregiver (Table 1). Enrolled children will be passively monitored to record both EPI vaccinations and PMC visits as well as clinical visits for malaria and anaemia to the health facility and/or CHWs over the 18-month study period. Older children (24 to 36 months at baseline) are not eligible to receive the intervention but will be enrolled in this study activity for two reasons: first, the older children will serve as an internal control as it is assumed that they will experience a similar malaria transmission intensity but will not receive the intervention and, secondly, as children will be followed for 18 months information will be available on children who age-out of eligibility to assess any impact beyond when children are eligible to receive PMC. Children will not be censored according to age.

During the passive cohort recruitment, trained fieldworkers will record basic demographic information about the child, recent malaria cases, amongst other information. An anonymous and unique QR code with the participant ID linked will be attached to each child’s health and vaccination record books. This QR code will be used to identify children participating in the study when they interact with the health system and facilitate linking the data generated by the routine system on EPI, PMC or clinical visit, which are stored in separate registers, with the research activities. During recruitment, the national health ID number assigned to each child and written in the health and/or vaccination record books will be recorded. The names of children and primary caregivers will also be recorded to provide a means to verify that children present match ID numbers. This also serves as a back-up method to link health information data that is routinely collected in different registers. Caregivers will be encouraged to take children to the participating facilities or CHWs to receive EPI vaccinations or PMC or for any illnesses.

Study staff will be present during the scheduled EPI visits to record when children received each dose of PMC. This may occur at health facilities or during active campaigns initiated by the health facility staff where vaccinations, including the newly introduced RTS, S vaccine where present, Vitamin A or SP is distributed. Data collected will include the child’s ID number, the date, location, and which health interventions were administered at that contact point. Where CHWs administer some of the SP or Vitamin A doses, they will record the data for the relevant contact point. The study staff will also collect, information on all clinical malaria and anaemia episodes throughout the 18-month study period. When a child participating in the passive cohort (i.e., has a QR code in their record book or the caregiver makes their participation known and confirmed through looking up their name) visits a study health facility or CHW, information about their visit will be recorded including date, diagnosis and treatment received and whether a referral was made. For children receiving inpatient treatment for anaemia or malaria, additional information including clinical symptoms and details of any complications, treatments received, the outcome, and date of discharge will be collected. Data will also be collected on any children who die in hospital during the study period. All data will be obtained from the clinical registries and confirmed by the facility staff.

Finally, to ensure that results are interpreted within the context of the broader trends in malaria transmission and the incidence of anaemia, routine surveillance data for each health facility will be collected up to five years before the start of the evaluation untill the end of the study. Data will consist of monthly aggregated data per health facility per 12-monthly age-band for children under 5 years of age. Key variables to be collected include the number of attendees, number of uncomplicated and complicated malaria cases, the number of malaria deaths, the number of anaemia and severe anaemia cases.

### Active cohort

The active cohort will measure malaria infection, anaemia, and malnutrition incidence in the intervention and control study populations in Cameroon and Côte d’Ivoire. The sampling frame for the active cohort will consist of the children whose primary caregiver provided consent for participation in the passive cohort (Table 1). Children between 6 and 9 months and 10 weeks and 6 months of age at recruitment in Cameroon and Côte d’Ivoire, respectively, will be approached for inclusion. Targeting these age ranges will maximize study power to measure differences between the PMC intervention and standard of care in each country within the 18-month study period. The target age range was also defined to ensure at least one sampling time point after the final potential PMC dose to observe any change in risk of malaria infection after aging out of the intervention [13]. Recruitment into the active cohort will continue until the target sample size is achieved.

At the initial household visit for children enrolled in the active cohort, field workers will record the household and child ID number assigned during the census and collect detailed information related to malaria risk factors including household construction and use of vector control interventions. A detailed questionnaire on recent fevers, malaria history, care seeking behaviours, EPI vaccines received to date, and other relevant factors will also be collected. Children will be assessed for fever, anaemia, malaria, and malnutrition. First, forehead temperature will be taken using an infrared thermometer with fever defined as those with temperature > 37.5 °C. A capillary blood sample by heel or fingerpick will be collected using a sterile lancet and aseptic technique on all children. The blood sample will be used to measure: (i) haemoglobin (Hb) levels with a photometer (HemoCue^®^ Hb 301, Angleholm, Sweden), devices, (ii) conduct a malaria rapid diagnostic test (RDT), (iii) prepare a thin and thick blood slide for malaria microscopy and (iv) to collect blood spots on filter paper. The mid-upper-arm circumference will be collected from all enrolled children to screen for malnutrition and monitor growth trajectories over time. Treatment for malaria, anaemia and malnutrition will be offered according to national guidelines. Any children with a fever or who are severely ill will be referred to the nearest health facility for treatment.

**Fig. 4:**
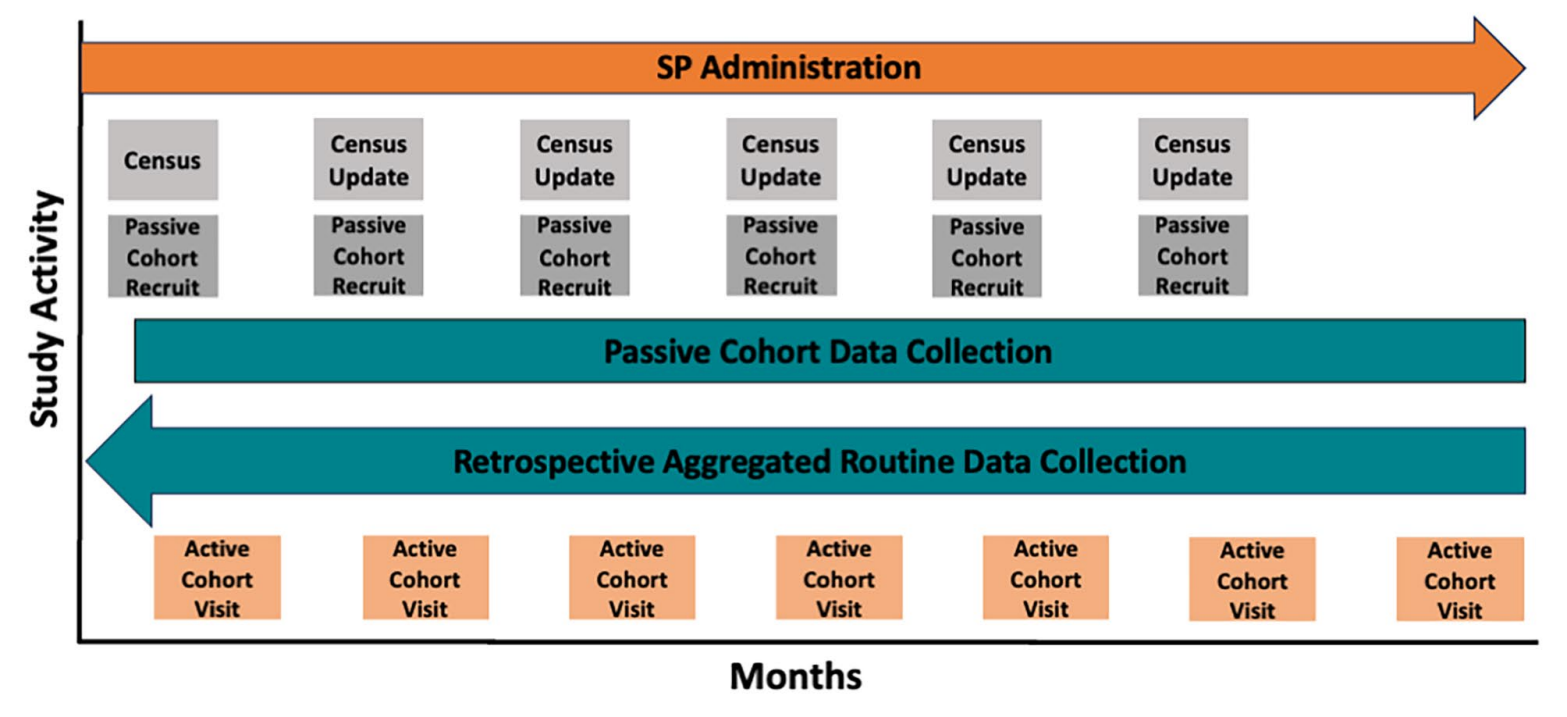
Title: Overview of impact evaluation study activities Legend: Timeline of the impact evaluation study activities including when the different study components take place and when they overlap

Each child in the active cohort will be followed-up for a maximum of seven visits at three-monthly intervals over 18 months, including the initial recruitment visit plus six subsequent follow-up visits (Fig. 4). During follow up visits at the household, verbal consent from the primary caregiver for continued participation of their child in the study will be recorded. A short questionnaire will be completed to collect data about any malaria or anaemia diagnosis or receipt of PMC since the last study visit, at which health facility care was sought and other relevant information. A blood sample will be collected following the same procedures outlined above.

Adverse events and safety of receiving PMC will be monitored by the MoH as part of the routine programmatic implementation of this intervention. Participant safety will be monitored throughout the study period and any adverse effects related to study procedures will be investigated using standardised procedures and managed according to national guidelines.

### Sample size

For the passive cohort, we will monitor all eligible children enrolled in the study and will therefore, will constitute a population sample. For the active cohort, the sample size was calculated to ensure 80% power at the 5% significance level, assuming an intra-cluster correlation coefficient of 0.25, and accounting for a 3:1 sampling ratio of intervention to control to ensure sufficient power to assess any variation in PMC effectiveness according to distance to the EPI facility. However, the specific sample size for the number of EPI facilities and children to include in the active cohort was calculated separately for each study site to account for the differences in PMC implementation (Fig. 1).

In Cameroon, we expect a 30% reduction in malaria incidence in intervention compared to control areas, requiring 26 intervention and 9 control EPI facilities are required. With a 20% expected loss to follow-up rate, we require 1,560 in the intervention and 540 children in the control area over the 18 months of observation in the active cohort. In Côte d’Ivoire, the standard of care currently being implemented by the NMP is no doses, so the difference between in the number of SP contacts between arms is 5. Therefore, we expect a 40% reduction in malaria incidence in intervention compared to control areas. Accordingly, 14 and 5 EPI facilities will be required in the intervention and control areas, respectively. Assuming a conservative loss to follow-up rate of 20%, we require 1,148 and 410 children in the intervention and control areas, respectively, over the 18 months of observation in the active cohort.

### Consenting procedures

During the census written informed consent will be obtained from the head of household and the primary caregiver of each eligible household and child. The potential participant will be reassured that refusal to participate or withdraw from the study at any point will have no impact on access to EPI vaccinations, PMC or routine health care. The head of household and/or primary caregiver will then be asked to sign the form as consent for their household and any eligible children to participate in the study, respectively. If the potential signatory is unable to sign, an independent adult witness not involved in the study, will sign on their behalf and the head of household or primary caregiver will provide a thumbprint marking their consent. If the signatory of a household/child is below the age of majority, defined as a person who is at least 21 in Cameroon and 18 years of age in Côte d’Ivoire, they will be asked to provide assent to confirm their willingness for their household/child to participate in the study in addition to consent from an appropriate person of legal age. A second consent form will be completed for permission for long-term storage and future use of the samples collected. Any amendments to the study protocol will be approved by all relevant ethics review boards and participants will provide additional consent, as described above, for the changes.

### Data analysis

The primary analysis will be based on intention to treat (ITT), including all children residing in the intervention area as the exposed group. All available data up to the time of censuring (e.g., loss to follow-up, withdrawal, death, or study completion), irrespective of whether the child received any, part, or all the intended PMC doses. An additional per-protocol analysis will be completed which includes children who received at least one dose, all doses, or specific doses of PMC. The period of protection provided by SP is up to one-month post treatment and thus will constitute the protective window of those receiving the intervention. Data for both countries will be combined for analysis as well as assessed separately to ensure context specific nuances are fully assessed.

Incidence rates for malaria and anaemia will be calculated using multivariable Poisson regression, difference-in-difference, and interrupted time-series models comparing intervention and control areas [14–16]. Incidence rates, rate differences, and rate ratios between intervention and control area will be calculated for the ITT and per-protocol analysis. Malaria incidence will be determined by the number of malaria cases confirmed by either RDT or microscopy identified in either the passive or active cohort. Person-time at risk for each study outcome will be calculated based on the date of recruitment into the study population until the date of censuring or study end. For children who leave the study prior to the end date, the date of exit will be the date where they moved, withdrew from the study, or died. If that date is not known, the midpoint between the date of last contact and the date the loss was detected will be used. Protective effectiveness will be calculated for the intervention period including the 18 months of follow-up for the periods where PMC doses differ, stratified by first and second year of life, as well as during the post-intervention period, starting at 24 or 18 months of age in Cameroon and Côte d’Ivoire, respectively, to estimate any potential rebound in malaria.

Where there are repeated measures per child, random effects or generalising estimating equations models will be used to adjust for non-independence of the data from the same individual. Regression models will assess factors associated with incidence of malaria infection including demographic variables, malaria history, reported vector control use, socio economic position, amongst others [17]. Transmission intensity, type of PMC delivery mode, impact of the estimated protective window of SP, and other factors will be adjusted for to assess their impact on the incidence trends [18]. Subgroup analysis will constitute assessing the impact of distance to the health facility as a proxy for access to health services according to the study endpoints highlighted above. Distance will be assessed according to tertials of distance of all study participants to their nearest health facility and as a continuous variable. Alternate model forms including interaction terms as well as semiparametric approaches to account for time-varying intervention effects to account for the expected 4-week prophylactic period of SP will be tested [19]. Because of the challenges in confirming cause of death in those who die in the community and the assumption that severe malaria cases will seek care at a health facility, only the passive cohort data will be used to assess these secondary outcomes [20]. An interim analysis will be conducted after the 4th active cohort visit and the study will be reported according to the ‘Strengthening the reporting of observational studies in epidemiology’ (STROBE) guidelines [21].

### Data collection and management

All data from the census, passive case detection and active case detection activities will be collected using electronic case record forms (eCRFs) using Open Data Kit (ODK [https://opendatakit.org]) and encrypted password protected Android devices. Completed eCRFs will be securely transferred to a 256-bit SSL encrypted and fire-walled ODK server, which is hosted at LSHTM [22]. We will design eCRFs in English and French languages to collect data on all aspects of the study, including, EPI vaccinations and SP distribution, longitudinal follow-up of children and all health facility visits. Automated programmes in R statistical software will push data to REDCap, software that is compliant with Good Clinical Practice for storing an audit trail of changes made during data cleaning, data management and further quality control checks [23]. Electronic data entry quality will be ensured by real-time error capture on ODK eCRFs, internal validation, consistency checks and stringent formatting constraints. The study protocol, standard operating procedures, data collection tools and other study documents will be freely available on request from coinvestigators and from the data repository website.

### Patient and public involvement and participant remuneration

The views of the relevant stakeholders were instrumental in developing the study protocol. Firstly, the intervention was designed during a co-creation workshop where stakeholders met to agree on the number, timing, and modality of SP doses as well as which areas would be included in the pilot implementation. Secondly, meetings with the clinical staff in the study areas as well as community leaders were held to provide input on the study procedures, including the eligibility criteria, piloting the questionnaire and other data collection mechanisms.

As is standard practice in both countries, study participants will not receive any financial renumeration for their participation. No payments for participants’ time or any costs associated with a routine health visit will be covered. As per the national strategy in both settings, all EPI, Vitamin A, and PMC services are free. For children participating in the active cohort all tests and any provided treatment will be provided at no cost to the participant.

### Dissemination of project findings

Dissemination of the study findings we will take multiple forms to maximise the reach of the information. Firstly, stakeholder meetings will take place within each study country where the interim and final results will be presented to inform programmatic decision-making on PMC implementation. Stakeholders will consist of the relevant NMP representative involved in policy as well as members of the community where the research took place so clinicians, CHWs, and the participants can see how their participation benefited research and translated into the results obtained. Peer-reviewed publications and presentations at national and international meetings will allow the results to be disseminated to the relevant scientific community. Authorship will follow the international committee of medical journal editors and equitable partnership guidelines. All papers will be submitted for publication in open access journals and anonymized data will be available for future research on institutional data repositories that can benefit the communities, where appropriate ethical approvals are in place.

## Discussion

Targeting malaria chemoprevention to those most at-risk of severe malaria has been identified as an important strategy to reduce morbidity and mortality in vulnerable populations. This is the first multi-country, large-scale population observational cohort study that will investigate the effectiveness of programmatically delivered PMC into the second year of life. This research will generate unique and well characterised population cohorts of children followed-up over 18-months in peri-urban and rural African settings. By combining multiple evaluation approaches including both passive and active cohorts, we will be able to address many policy-relevant and implementation questions. Our methodological innovations include use of unique ID numbers per child in both the passive and active cohorts which enables collection and linking of data across multiple health services including EPI vaccination visits, treatment seeking episodes, and any active cohort visits. Similarly, the inclusion of monitoring the older children, beyond the age of PMC eligibility, ensures an internal control as well as monitor the potential impact of PMC on malaria as children age out of the programme [24]. The current study builds on the growing evidence of how chemoprevention strategies can reduce malaria morbidity and mortality when targeted to high-risk populations and can be integrated into routine health services to facilitate rapid scale-up [5]. The participatory approach to research design as well as strong collaborations with national and international policy makers will ensure that the study results are relevant and can be translated widely to inform national policy and practice.

Although PMC is a WHO recommended strategy, there are several gaps in the current evidence that if filled, would help support how, when, and where to implement this strategy to achieve maximum impact. Firstly, the available malaria interventions targeting a reduction in morbidity and mortality, including the recently licensed vaccine, are imperfect and thus only confer partial protection [10, 25]. Therefore, NMPs are faced with having to make decisions on which interventions to invest in, targeted to which populations, where and when. Understanding how the additional doses of SP over the first two years of life impacts malaria burden as well as important nuances including which doses confer more protection and why (e.g., low coverage at a given EPI contact point, administered in the low vs. high malaria transmission season etc.) will provide needed evidence to help justify and optimise PMC implementation [9]. Secondly, controlled trials are essential for generating evidence to inform policy changes and subsequent recommendations for given interventions. However, when such interventions are implemented in a programmatic setting, they are unlikely to achieve the same degree of impact as when done in a controlled setting. Our study will address this specific gap and directly measure the programmatic effectiveness of PMC with SP while the robust study design allowing in depth analysis of key questions including any dose-response effect, which doses maximizes impact, amongst others. This evaluation using real-world data, will therefore, fill important knowledge gaps while simultaneously supporting broader analysis on cost-effectiveness of the intervention or the implications of parasite drug-resistance profiles on the protective effectiveness of SP.

In conclusion, our research team will undertake a comprehensive and robust evaluation of the effectiveness of PMC to reduce malaria burden. Global trends in malaria have had recent trends in reducing burden stagnating or in some cases, increasing. Therefore, new evidence-based strategies are urgently needed to expand the arsenal of malaria control tools available. The participatory and inclusive nature of the study design and broader research collaboration ensures the potential for the findings to be widely implemented by other NMPs wanting to expand their arsenal of options available to help reveres these trends.

## Electronic supplementary material

Below is the link to the electronic supplementary material.

**Supplementary file 1**: Model information sheets and consent forms for each study element.

Supplementary Material 2

## Data Availability

No datasets were generated or analysed during the current study.

## Abbreviations

CHW: Community Health Worker
CI: Confidence Interval
eCRF: Electronic Case Record Form
EPI: Essential Programme of Immunization
Hb: Haemoglobin
IPTi: Intermittent Preventative Treatment for malaria in Infants
ITT: Intention to Treat
NMP: National Malaria Programme
MoH: Ministry of Health
ODK: Open Data Kit
PMC: Perennial Malaria Chemoprevention
RDT: Rapid Diagnostic Test
SP: Sulfadoxine-Pyrimethamine
WHO: World Health Organization
