# Supplementary material for: Effectiveness of malaria chemoprevention in the first two years of life in Cameroon and Côte d’Ivoire compared to standard of care: study protocol for a population-based prospective cohort impact evaluation study
Source: BMC Public Health. 2024 Sep 6;24:2430. doi: 10.1186/s12889-024-19887-8 (PMC11378462; doi:10.1186/s12889-024-19887-8)
Supplement: Supplementary file 1 — Model information sheets and consent forms for each study element. [file 12889_2024_19887_MOESM1_ESM.docx]

**Supplementary File 1: Model information sheets and consent forms for each study element**

Table of Contents

[Census – Participant Information Sheet 2](#_Toc168487027)

[Census - Householder Informed Consent Form 4](#_Toc168487028)

[Passive Cohort – Participant’s Information Sheet 5](#_Toc168487029)

[Passive Cohort – Informed Consent Form 8](#_Toc168487030)

[Passive Cohort – Informed Assent Form 9](#_Toc168487031)

[Active Cohort - Information Sheet Recruitment Visit 10](#_Toc168487032)

[Active Cohort – Informed Consent Form 13](#_Toc168487033)

[Future Use of Samples - Information Sheet 14](#_Toc168487034)

[Future Use of Samples - Informed Consent Form 15](#_Toc168487035)

# **Census – Participant Information Sheet**

SP/IPTi+ Impact Assessment: Census

**Investigators and institutions involved:**

*London School of Hygiene and Tropical Medicine* (LSHTM) – London, UK

*University of South Florida, USA*

*FI-CHITRES*

**Funder**: UNITAID

**Introduction**

We would like to your household to take part in a research study about malaria in the community. Taking part in the study is entirely up to you. Before you decide, you should understand why the research is being done and what it involves. Someone from our research team will review this information sheet with you and answer any questions you may have. Please do ask questions if anything is not clear, or if you would like more information. Please feel free to talk to others about the study if you wish. It is fine to take time to decide whether or not to take part. We will discuss the study together and give you a copy of this information sheet. If you agree for your household to take part, we will then ask you to sign a consent form.

**What is the purpose of the study?**

Researchers involved in the study are interested in learning more about preventing malaria in young children in Africa. The malaria programmes in Cameroon are starting a preventative treatment programme to provide young children with extra doses of a malaria drug called sulfadoxine-pyrimethamine, or SP, because we think that it helps to prevent malaria and anaemia in in children under two years of age, but it is not known if giving SP during the second year has benefits protecting children from malaria. This study will help to find out whether the programme being implemented by the Ministry of Health where they are trialing giving SP to older children helps protect children that are eligible for the programme.

**Does my household have to take part?**

Your household does not have to take part. It is up to you to decide if your household is to participate. If you decide not to take part, we will not collect any information from you.

**What will happen if my household takes part?**

If you agree to have your household participate in the study, we will collect some information on your household, how many people live there, their age, gender and some information about malaria control. We will give your household a unique identification number, and record the geographic location so that we can find it in the future and update your household information. We would also like to record the names of the children and their primary caregivers. All names will be removed from the database once it is finalized, and it will not be revealed in any documents or publications or websites. This census will take approximately 20 minutes, and we will return every four months over the next two years to make sure that the information is still correct.

**What are the possible risks and disadvantages?**

Participation in any research study may involve a loss of privacy. Information about your household will be collected as part of the study, but only the people working on the study will see it. We will use a study number, rather than your name, to identify your information. Your name will not be written in any reports based on this research.

**What are the possible benefits?**

The knowledge gained from this study will help researchers and policymakers understand how best to prevent malaria in this area for children in the future.

**What if something goes wrong?**

If you have a concern about any aspect of this study, you should ask to speak to the staff who will do their best to answer your questions. The research team in Cameroon can be contacted at XXXXX. If the research team cannot address your concerns, and you wish to complain formally, you can do this by contacting LSHTM staff member XXXXX. The London School of Hygiene and Tropical Medicine holds insurance policies which apply to this study. If your household experiences harm or injury as a result of taking part in this study, you may be eligible to claim compensation.

**Can I change my mind about my household taking part?**

Yes. You can withdraw your household from the study by telling research team member or when you visit the health provider. If you do decide to withdraw your household from the study, we will ask your permission as to whether we can use the information collected before your household stopped taking part.

**What will happen to information collected about my household?**

All information collected about your household will be kept private. Only the study staff and authorities who check that the study is being carried out properly will be allowed to look at information about your household. Any information about your household will be anonymised this means all information will be identifiable only using a unique identification number. The exact location of you household will not be accurate so no one will be able to identify your exact household.

Information will be made available to other researchers worldwide for research that is important to Cameroon and to improve medical knowledge and patient care. Your household’s personal information will not be included and there is no way that the household can be identified.

**What will happen to the results of this study?**

The study results will be published in medical journals so that health workers, public health specialists and other researchers can learn from them. We will present the results to the Ministries of Health and other authorities in the participating countries. Name and personal information will not be included in any study reports.

**Who is organising and funding this study?**

This study is funded by Unitaid. London School of Hygiene & Tropical Medicine is the sponsor for the research, and they have full responsibility for the project including the collection, storage and analysis of data from your household.

**Who has checked this study?**

This study has been checked by an independent group of people, called a Research Ethics Committees, for fairness of the study procedures, accuracy and transparency. This study was reviewed and approved by the following ethics committees: the National Ethics Committee in Cameroon, the Comité Consultatif National de Bioéthique de la Répuublique de Côte d’Ivoire, the World Health Organization in Switzerland, and the London School of Hygiene and Tropical Medicine in the United Kingdom. And they have agreed that it is OK for us to ask people to take part.

**Whom can I talk with if I have questions about participating, before, during or after the study?**

You are welcome to contact the research team at any time. The research team in Cameroon can be contacted at XXXXXX.

**Providing consent to participate in the study**

Thank you for taking time to review this information. If you would like your household to take part in the study please read and sign the consent form on the next page.

**
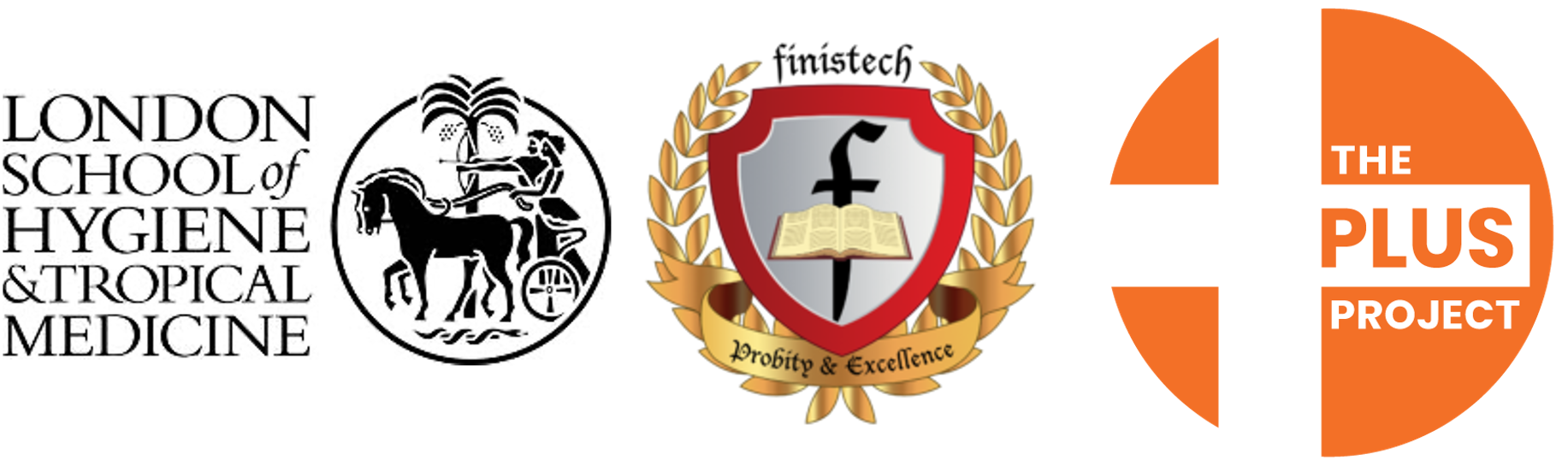
****Study title: Evaluation of the impact of SP/TPIn+.**

# **Census - Householder Informed Consent Form**

**HH ID: CT00001HH**


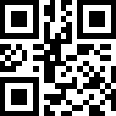
**Declaration Please affix your initials or . imprint on each square**

The study staff explained the information to me in a language I could understand. I had the opportunity to review the information, ask questions and get satisfactory answers.

I understand that my participation is voluntary and that I am free to withdraw at any time without giving any reason, without this affecting my medical care or legal rights.

I understand that relevant sections of my data collected during the course of the study may be accessed by authorized individuals from the research team and monitoring agencies, where relevant to my participation in this research. I authorize these persons to access my files.

I understand that my data will be shared through a public data repository or by sharing it directly with other researchers, and that I will not be identifiable from this information.

I agree to take part in this malaria study.

**Print name of head of household Signature or imprint of thumb**

training

**Current date**

**(DD/MM/YYYY)**

|  |  |  |
| --- | --- | --- |

**Printed name of impartial witness Signature of witness Current date**

**(DD/MM/YYYY)**

|  |  |  |
| --- | --- | --- |

**I certify that I have accurately explained the study information, that it has been understood to the best of my knowledge and that they have freely given their consent to participate in the presence of the impartial witness named above (if applicable).**

**Printed name of person obtaining consent**

**Signature of person Current date**

**(DD/MM/YYYY)**

|  |  |  |
| --- | --- | --- |

# **Passive Cohort – Participant’s Information Sheet**

SP/IPTi+ Impact Assesment: Passive Cohort

**Investigateurs et institutions impliquées :**

**London School of Hygiene and Tropical Medicine – London, UK (LSHTM)**

**University of South Florida, USA**

**FI-CHITRES – Yaoundé, Cameroun**

**Funder : UNITAID**

**Introduction**

We would like to invite your children under 3 years old (less than 36 months) to take part in a research study. Having children who live at this household join the study is entirely up to you. Before you decide, you should understand why the research is being done and what it involves. Someone from our research team will review this information sheet with you and answer any questions you may have. Please do ask questions if anything is not clear, or if you would like more information. Please feel free to talk to others about the study if you wish. It is fine to take time to decide whether or not to take part. We will discuss the study together and give you a copy of this information sheet. If you agree for your child to take part, we will then ask you to sign a consent form.

**What is the purpose of the study?**

Researchers involved in the study are interested in learning more about preventing malaria in young children in Africa. The malaria programmes in Cameroon are starting a preventative treatment programme to provide young children with extra doses of a malaria drug called sulfadoxine- pyrimethamine, or SP, because we think that it helps to prevent malaria and anaemia in young children, but it is not known if giving SP during the second year has benefits protecting children from malaria. This study will help to find out whether the programme being implemented by the Ministry of Health where they are trialling giving SP to older children helps protect children that are eligible for the programme.

**Does my child have to participate?**

Your child does not have to take part. It is up to you to decide if your child participates. The health workers will still care for your child according to national medical guidelines and they will be offered the malaria drug during routine immunisation visits, even if your child does not take part in this research study, and your decision will not affect the quality of care your child receives now or in the future.

**What will happen if my child participates?**

If you agree to have your child children participate in the study, we will collect some information on your household, assign a unique identification number, and record the geographic location. For all children residing in this household that are under 36 months old, we would collect more detailed information on them including their age, gender, and how many times they have had malaria and other related questions. We would also like to record the name of the child and of their parents in case we need to follow-up with you during the study. All names will be removed from the database once it is finalized, and it will not be revealed in any documents or publications or websites.

We will assign each child a unique identification number connected to a barcode that will be attached to their health book. This barcode will help us keep track of every time your child visits the health facility to get a vaccination, vitamin A, or malaria treatment. If your child gets sick with malaria or anaemia over the study period, we will also use this barcode to record how many times this happens. For each visit to a health provider participating in the study when malaria or anaemia is diagnosed, we will collect some information about the illness including if the child had to be admitted to the hospital or the health facility, and if so, for how long, the symptoms your child experienced, what treatment they received and other information routinely collected by the health worker.

You and your child may also be chosen by a lottery for another follow-up study. In this other study the study team will visit you again and ask if you wish to be a part of the follow-up study.

**What are the possible risks and disadvantages?**

Participation in any research study may involve a loss of privacy. We will collect details about your household and children, including recording visits for health care. Personal details and information about your child’s illness will be collected as part of the study, but only the people working on the study will see it. We will use a study number connected to the barcode your child receives to identify the data collected. Your or your child’s name will not be written in any reports based on this research.

**What are the possible benefits?**

Whether or not you decide for your child to take part in the study, your child will receive standard care from health providers in your community. No payment will be provided for your child’s participation or to support them in seeking routine health treatment. The knowledge gained from this study will help researchers and policymakers understand how best to prevent malaria in this area for children in the future.

**What if something goes wrong?**

If you have a concern about this study, you should ask to speak to the staff who will do their best to answer your questions. The research team in Cameroon can be contacted at XXXXX. If the research team cannot address your concerns, and you wish to complain formally, you can do this by contacting [LSHTM staff member XXXX. The London School of Hygiene and Tropical Medicine holds insurance policies which apply to this study. If your household experiences harm or injury as a result of taking part in this study, you may be eligible to claim compensation.

**Can I change my mind about my child's participation?**

Yes. You can withdraw your child from the study by telling research team member when you visit the health provider. The health workers at the clinic will still provide care and treat your child with the national medical guidelines. If you do decide to withdraw your child from the study, we will ask your permission as to whether we can use the information collected before your child stopped taking part.

**What will happen to the information collected about my child?**

All information collected about your child will be kept private. Only the study staff and authorities who check that the study is being carried out properly will be allowed to look at information about your child. Any information about you or your child will be anonymised this means all information will be identifiable only using a unique identification number.

Information about your child will be stored securely by the study team. Their personal details will be kept in a different safe place to the other study information and will be destroyed once the database is finalized. At this point, your child will only be identified using the unique identification number. At the end of the project, the study data will be archived locally and in London.

Information will be made available to other researchers worldwide for research that is important to Cameroon and to improve medical knowledge and patient care. You or child’s personal information will not be included and there is no way that you or your child can be identified.

**What will happen to the results of this study?**

The study results will be published in medical journals so that health workers, public health specialists and other researchers can learn from them. We will present the results to the Ministries of Health and other authorities in the participating countries. Your child’s name or their personal information will not be included in any study reports.

**Who is organising and funding this study?**

This study is funded by Unitaid. The London School of Hygiene & Tropical Medicine is the research sponsor and takes full responsibility for the project, including the collection, storage and analysis of your child's data.

**Who has checked this study?**

This study has been checked by an independent group of people, called a Research Ethics Committees, for fairness of the study procedures, accuracy and transparency. This study was reviewed and approved by the following ethics committees: the National Ethics Committee in Cameroon, the Comité Consultatif National de Bioéthique de la République de Côte d’Ivoire, the World Health Organization in Switzerland, and the London School of Hygiene and Tropical Medicine in the United Kingdom. And they have agreed that it is OK for us to ask people to take part.

**Who can I contact if I have questions about participation, before, during or after the study?**

You are welcome to contact the research team at any time. The research team in Cameroon can be contacted at XXXX.

**Give consent to participate in the study**

Thank you for taking the time to read this information. If you would like your child to participate in the study, please read and sign the consent form on the next page.

# **Passive Cohort – Informed Consent Form**

**Study Title:** SP/IPTi+ Impact Evaluation: Passive cohort

| **House Code:** |  | **Person Code:** |  | **Date (dd/mm/yyyy):** | / / |
| --- | --- | --- | --- | --- | --- |
| **Childs Full Name:** |  | | | | |

| **Statement** | **Please initial or thumbprint** |
| --- | --- |
| I have had the information explained to by study personnel in a language that I understand. I have had the opportunity to consider the information, ask questions and have these answered satisfactorily. |  |
| I understand that my child’s participation is voluntary and that my child are free to withdraw at any time without giving any reason, without their medical care or legal rights being affected. |  |
| I understand that relevant sections of my child’s data collected during the study may be looked at by authorised individuals from the research team and oversight bodies, where it is relevant to my taking part in this research. I give permission for these individuals to have access to my child’s records. |  |
| I understand that data about my child may be shared via a public data repository or by sharing directly with other researchers, and that my child will not be identifiable from this information. |  |
| I agree for my child to take part in this malaria study. |  |

Printed name of parent/guardian Signature or thumb print of parent/guardian Date

Printed name of impartial witness Signature of impartial witness Date

I attest that I have explained the study information accurately, and it was understood to the best of my knowledge by the parent/guardian and that he/she has freely given their consent for the child to participate* in the presence of the impartial witness named above (where applicable).

Printed name of person obtaining consent Signature of person obtaining consent Date

# **Passive Cohort – Informed Assent Form**

Study Title: SP/IPTi+ Impact Evaluation: Passive cohort

| **House Code:** |  | **Person Code:** |  | **Date (dd/mm/yyyy):** | / / |
| --- | --- | --- | --- | --- | --- |
| **Childs Full Name:** |  | | | | |

| **Statement** | **Please initial or thumbprint* each box** |
| --- | --- |
| I have had the information explained to by study personnel in a language that I understand. I have had the opportunity to consider the information, ask questions and have these answered satisfactorily. |  |
| I understand that my child’s participation is voluntary and that my child are free to withdraw at any time without giving any reason, without their medical care or legal rights being affected. |  |
| I understand that relevant sections of my child’s data collected during the study may be looked at by authorised individuals from the research team and oversight bodies, where it is relevant to my taking part in this research. I give permission for these individuals to have access to my child’s records. |  |
| I understand that data about my child may be shared via a public data repository or by sharing directly with other researchers, and that my child will not be identifiable from this information. |  |
| I agree for my child to take part in this malaria study. |  |

Printed name of parent/guardian Signature or thumb print of parent/guardian Date

Printed name of impartial witness Signature of impartial witness Date

I attest that I have explained the study information accurately, and it was understood to the best of my knowledge by the parent/guardian and that he/she has freely given their consent for the child to participate* in the presence of the impartial witness named above (where applicable).

Printed name of person obtaining consent Signature of person obtaining consent Date


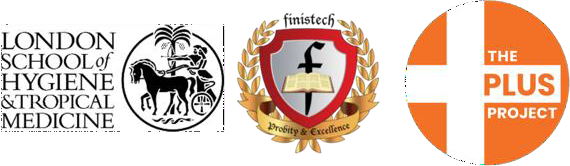


# **Active Cohort - Information Sheet Recruitment Visit**

**For Parents or Guardians of Children**

**Study Title:** SP/IPTi+ Impact Evaluation: Cohort

**Investigators and Institutions Involved:**

*London School of Hygiene & Tropical Medicine* (LSHTM)

*Fobang Institutes-CHITRES – Cameroon*

**Funder**: UNITAID

**Introduction**

You recently agreed for your child to participate in our research study where we are recording their visits to receive routine vaccinations and medicines as well as any diagnosis of malaria or anaemia by a health provider. We have selected your child to participate in the more detailed research study that we would like your child to take part in. This study would involve our study team visiting every three months over the next 1.5 years to ask some questions and test your child for malaria and anaemia. Having your child join this part of the study is entirely up to you. Before you decide, you should understand why the research is being done and what it involves. Someone from our research team will review this information sheet with you and answer any questions you may have. Please do ask questions if anything is not clear, or if you would like more information. Please feel free to talk to others about the study if you wish. It is fine to take time to decide whether or not to take part. We will discuss the study together and give you a copy of this information sheet. If you agree for your child to take part, we will then ask you to sign a consent form.

**What is the purpose of the study?**

Researchers involved in the study are interested in learning more about preventing malaria in young children in Africa. The malaria programmes in Cameroon and Cote d’Ivoire are implementing a programme to provide young children with extra doses of a malaria drug called sulfadoxine-pyrimethamine, or SP because we think that it helps to prevent malaria and anaemia in in children under two years of age. The World Health Organization recommends the use of SP among children up to one to prevent malaria, but it is not known if giving SP through the second year of life has a bigger impact on protecting children from malaria. This study will help to find out how much protection the additional doses of SP gives against malaria and anaemia including which of the scheduled doses may be more important.

**Does my child have to take part?**

Your child does not have to take part. It is up to you to decide if your child is to participate. Your child will still receive all care normally received by health providers even if your child/children does not take part in this study, and your decision will not affect the quality of care your child receives now or in the future.

**What will happen if my child takes part?**

If you agree to have your child participate in the study, we will collect some additional information on your household and child than we collected the last time a study team was here. During this first visit we would collect more detailed information including what kind of things you do to protect your child against mosquito bites, how regularly you follow the vaccination schedule, for example. We will measure your child’s temperature and ask if they have had a fever in the last two days, measure the size of your child’s arm to monitor their growth, and take a blood sample from your child’s heel if 3 months of age or younger or their finger if older than 3 months to examine for malaria parasites and test the blood levels to see if they have anaemia. We will use a small needle to make the prick on finger, and the total amount of blood taken will the less than half a teaspoon (2ml) to do a malaria rapid diagnostic test (RDT) and put onto a filter paper. The filter paper will be stored in a laboratory and used to do extra tests to better understand malaria. If the rapid test is positive, we will give your child the first-line treatment that is policy in Cameroon/Cote d’Ivoire. If your child has low blood levels, we will provide first-line treatment or iron supplements following the national policies. All the medicines used in the study have been approved by the Cameroon/Cote d’Ivoire Ministry of Health. You can bring your child for care even when there is no scheduled visit and clinical staff will test and treat your child if he or she has symptoms of malaria.

We will return to visit your child every three months over the next year and a half. During these six more visits (seven in total, including today), we will ask you to give verbal confirmation that you consent to your child continuing to participate in the study. We will ask a small number of questions to find out if they have been diagnosed with malaria and which vaccines they have received since the previous visit and collect another blood sample to test for malaria by RDT, anaemia, and to collect the sample on filter paper. Anyone testing positive will receive treatment according to the national standard.

All information will be connected to the same identification number and barcode they were assigned the last time our teams were here. If your child feels unwell between our study visits, we encourage you to take them to one of the participating facilities for assessment and treatment. Your child participating in this more detailed study or not, does not affect the other study procedures where we record the details of visits to the vaccination clinic or any malaria or anaemia diagnoses.

**What are the possible risks and disadvantages?**

The risks of drawing blood from a heel or finger prick include temporary discomfort from the needle stick, bruising, skin infection and fainting. The amount of blood removed will be too small to affect your child’s health and the techniques our teams use make these risks as small as possible. There are also risks about the information we are collecting and we will collect details about your household and children, including recording visits for health care. Personal details and information about your child’s illness will be collected as part of the study, but only the people working on the study will see it. We will use a study number connected to the barcode your child receives to identify the data collected. You or your child’s name will not be written in any reports based on this research, and it will not be revealed in any documents or publications or websites.

**What are the possible benefits?**

Whether or not you decide for your child to take part in the study, your child will receive standard care from health providers in your community. As part of this survey, we will test your child for malaria and anaemia and if positive we will give your child appropriate treatment free of charge. However, we will not provide payments for your child participating in this study. The knowledge gained from this study will help researchers and policymakers understand how best to prevent malaria in this area for children in the future.

**What if something goes wrong?**

If you have a concern about any aspect of this study, you should ask to speak to the staff who will do their best to answer your questions. The research team in Cameroon can be contacted at XXXX, if the research team cannot address your concerns, and you wish to complain formally, you can do this by contacting [LSHTM staff member XXXX at XXXX]. The London School of Hygiene and Tropical Medicine holds insurance policies which apply to this study. If your child experiences harm or injury as a result of taking part in this study, you may be eligible to claim compensation.

**Can I change my mind about my child taking part?**

Yes. You can withdraw your child from the study at any time by telling research team member when you visit the health provider or the team visits your household. The health workers at the clinic will still provide standard care for your child. If you do decide to withdraw your child from the study, information that you provided before your child stopped taking part will still be used.

**What will happen to information collected about my child?**

All information collected about your child will be kept private. Only the study staff and authorities who check that the study is being carried out properly will be allowed to look at information about your child. Data will be sent to other study staff in Cameroon/Cote d’Ivoire and overseas but this will be anonymised. This means that any information about your child which is collected will be identifiable only using a unique identification number.

Information about your child will be stored securely by the study team. Her/his personal details will be kept in a different safe place to the other study information and will be destroyed once the database is finalized. At this point, your child will only be identified using the unique identification number. At the end of the project, the study data will be archived locally and in London. The data will be made available to other researchers worldwide for research that is important to the Ministry of Health in Cameroon/Cote d’Ivoire and to improve medical knowledge and patient care. Your child’s personal information will not be included and there is no way that s/he can be identified.

**What will happen to the results of this study?**

The study results will be published in medical journals so that health workers, public health specialists and other researchers can learn from them. We will present the results to the Ministries of Health and other authorities in the participating countries. Your child’s name and personal information will not be included in any study reports.

**Who is organising and funding this study?**

This study is funded by Unitaid. London School of Hygiene & Tropical Medicine is the sponsor for the research and they have full responsibility for the project including the collection, storage and analysis of data from your child.

**Who has checked this study?**

All research involving human participants is looked at by an independent group of people, called a Research Ethics Committee, to protect your child’s interests. This study has been reviewed and approved by the following ethics committees: the National Ethics Committee in Cameroon, the Comité Consultatif National de Bioéthique de la République de Côte d’Ivoire, the World Health Organization in Switzerland, and the London School of Hygiene and Tropical Medicine in the United Kingdom.

**Whom can I talk with if I have questions about participating, before, during or after the study?**

You are welcome to contact the research team at any time. Below is the contact information of the local investigators.

Cameroon: XXXX at XXXX

**Providing consent to participate in the study**

Thank you for taking time to review this information. If you would like your child to take part in the study please read and sign the consent form on the next page.


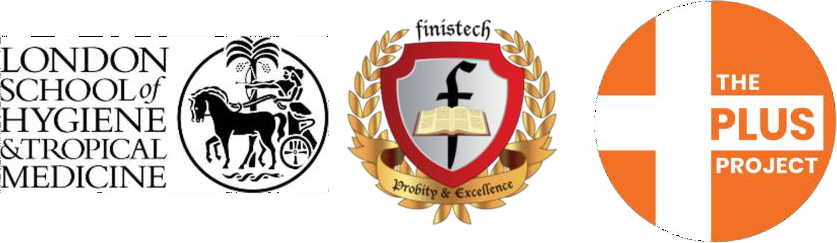


# **Active Cohort – Informed Consent Form**

Study Title: SP/IPTi+ Impact Evaluation: Active cohort

| **[Household QR Code]** |  | **[Child Code QR]** |  | Date (DD/MM/YYYYY):  / / |
| --- | --- | --- | --- | --- |
| Full name of child | | | | |

| **Statement** | **Please initial or**  **thumbprint* each box** |
| --- | --- |
| The study staff explained the information to me in a language I understand. I had the opportunity to review the information, ask questions and get satisfactory  answers. |  |
| I understand that my child's participation is voluntary and that they are free to  withdraw at any time without giving a reason, without their medical care or legal rights being affected. |  |
| I understand that relevant sections of my child's data collected during the study may be viewed by authorized individuals from the research team and oversight bodies, where relevant to my participation in this research. I give these people  permission to access my child's records. |  |
| I understand that blood samples taken from my child for this research study may  be sent to overseas laboratories, including the UK, for analysis. |  |
| I understand that data about my child may be shared through a public data  repository or by sharing it directly with other researchers, and that my child will not be identifiable from this information. |  |
| I agree for my child to take part in this malaria study. |  |

| Printed name of parent/guardian | Signature or thumb print of parent/guardian | Date (DD/MM/YYYYY) |
| --- | --- | --- |
|  |  |  |

| Printed name of impartial witness | Signature of impartial witness | Date (DD/MM/YYYYY) |
| --- | --- | --- |
|  |  |  |

I attest that I have explained the study information accurately, and it was understood to the best of my

knowledge by the parent/guardian and that he/she has freely given their consent for the child to participate* in the presence of the impartial witness named above (where applicable)

| Printed name of person obtaining  consent | Signature of person obtaining consent | Date (DD/MM/YYYYY) |
| --- | --- | --- |
|  |  |  |


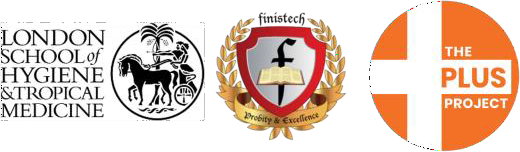


# **Future Use of Samples - Information Sheet**

**Introduction**

While your child is in this study, we mentioned that we will collect samples of her/his blood on a filter paper. This sample may be useful for future research. These samples will be stored for a long time. Samples may also be shared with other researchers who work directly with the research team.

**What will be done with my child’s samples?**

The samples from each visit will be used to study malaria infection. Research on these samples will happen in the future, and any information we get from these studies will not affect your child’s care. Your child’s samples will be used only for research. They will not be sold or used to make a profit or to make money.

**Will my child’s samples be stored confidentially?**

The samples will be identified only by study numbers and codes; they will not be labelled with your child’s name or any personal information. We will not put reports about research done with your child’s samples into your medical record.

**What are the risks and benefits of storing my child’s samples for future use?**

There are no known risks to you from future use of your child’s samples. There will be no direct benefit to you or your child from any future research on stored samples either. From studying samples from your child, we may learn more about infections that cause fever. We may learn how to prevent them, how to treat them, or how to cure them.

**May I change my mind?**

If you agree today to allow your child’s samples to be stored for future use, you may change your mind at any time. If you do change your mind, simply contact the study team. We will make sure samples from your child are destroyed and are no longer used for future research. Your child is still welcome to participate in the study if you decide today that you do not want us to store samples for future use.

**Providing consent for future use of biological samples**

Thank you for taking time to read this information leaflet. If you would like your child’s samples to be stored for future research, please read and sign the consent form on the next page.


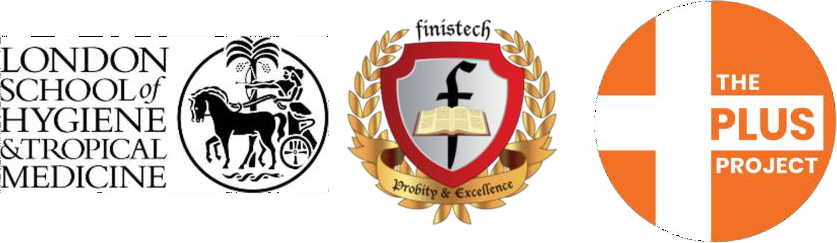


# **Future Use of Samples - Informed Consent Form**

Study Title: SP/IPTi+ Impact Evaluation: Active cohort

| **[Household QR Code]** |  | **[Child Code QR]** |  | Date (DD/MM/YYYYY):  / / |
| --- | --- | --- | --- | --- |
| Full name of child | | | | |

| **Statement** | **Please initial or**  **thumbprint* each box** |
| --- | --- |
| The sample storage information was explained to me by the study staff in a language I understand. I had the opportunity to review the information, ask questions and get satisfactory answers. |  |
| I understand that sample storage is voluntary and that I am free to withdraw my child's samples at any time without giving a reason, without affecting my child's medical care or legal rights. |  |
| I understand that samples taken from my child will be used to support further research in the future, and may be shared anonymously with other researchers in different countries, for their ethically approved projects of public health significance by the Ministry of Health. |  |
| I agree that my child's samples will be kept for future research. |  |

| Printed name of parent/guardian | Signature or thumb print of parent/guardian | Date (DD/MM/YYYYY) |
| --- | --- | --- |
|  |  |  |

| Printed name of impartial witness | Signature of impartial witness | Date (DD/MM/YYYYY) |
| --- | --- | --- |
|  |  |  |

I attest that I have explained the study information accurately, and it was understood to the best of my knowledge by the parent/guardian and that he/she has freely given their consent for the child to participate* in the presence of the impartial witness named above (where applicable)

| Printed name of person obtaining  consent | Signature of person obtaining consent | Date (DD/MM/YYYYY) |
| --- | --- | --- |
|  |  |  |
